# Dietary Composition and Its Association with Newly Diagnosed Nonalcoholic Fatty Liver Disease and Insulin Resistance

**DOI:** 10.3390/nu13124438

**Published:** 2021-12-11

**Authors:** Phunchai Charatcharoenwitthaya, Eakchakarj Tansakul, Kusuma Chaiyasoot, Wimolrak Bandidniyamanon, Natthinee Charatcharoenwitthaya

**Affiliations:** 1 Department of Medicine, Division of Gastroenterology, Faculty of Medicine Siriraj Hospital, Mahidol University, Bangkok 10700, Thailand; eakchakarj@gmail.com (E.T.); brink_amm_n@hotmail.com (W.B.); 2 Department of Medicine, Division of Clinical Nutrition, Faculty of Medicine Siriraj Hospital, Mahidol University, Bangkok 10700, Thailand; kusuma.chs@mahidol.ac.th; 3 Department of Medicine, Division of Endocrinology, Faculty of Medicine, Thammasat University, Pathum Thani 12121, Thailand; natthineei@yahoo.com

**Keywords:** nonalcoholic fatty liver disease, protein, full-fat dairy product, dietary fiber, vitamin D deficiency

## Abstract

Dietary modification is essential for treating nonalcoholic fatty liver disease (NAFLD); however, the dietary components are less well defined. We enrolled 252 adults with no history of liver disease and excessive alcohol use to evaluate the relationship between macronutrients and NAFLD and insulin resistance. Participants took photographs of their meals and documented their food intake in a food diary for seven consecutive days. A dietitian estimated the type and portion size of food items and analyzed nutrients with INMUCAL-Nutrients software. Later, participants underwent transient elastography to diagnose NAFLD and blood tests to measure insulin resistance using the homeostasis model. Total energy intake and the proportion of carbohydrate, fat, and protein consumption did not differ between participants with NAFLD (*n* = 41) and those without NAFLD (*n* = 211). Using multiple logistic regression analysis, daily intake of protein < 1.0 g/kg (OR: 3.66, 95% CI: 1.41–9.52) and full-fat dairy product ≥ 50 g (OR: 0.42, 95% CI: 0.18–0.99) were associated with NAFLD. Insulin resistance was associated with a daily intake of protein < 1.0 g/kg (OR: 3.09, 95% CI: 1.59–6.05), full-fat dairy product ≥ 50 g (OR: 0.46, 95% CI: 0.25–0.82), and dietary fiber ≥ 8 g (OR: 0.41, 95% CI: 0.22–0.74). Our data show that a low protein intake increases the odds for NAFLD and insulin resistance. Contrarily, a high intake of full-fat dairy products and dietary fiber has been associated with a potential protective effect against NAFLD and insulin resistance.

## 1. Introduction

Nonalcoholic fatty liver disease (NAFLD) has become the most prevalent chronic liver disease in the world, with an estimated prevalence of 25% among adults [1]. The rise in NAFLD prevalence is linked to the contemporary epidemics of obesity, Western-type dietary pattern, and sedentary lifestyle [2]. NAFLD can progress to more severe liver disease with liver fibrosis or cirrhosis, which can eventually lead to liver-related death [3]. Furthermore, insulin resistance and the different features of metabolic syndrome were associated with the development and progression of NAFLD, even in the absence of obesity and diabetes [4]. Clinical evidence shows NAFLD to be associated with liver-related morbidity and mortality and an increased risk of developing important extrahepatic chronic diseases, such as cardiovascular disease, diabetes, and chronic kidney disease [5]. Currently, the cornerstone of NAFLD management is lifestyle modification. Changes in lifestyle factors include a well-balanced diet and adequate physical activity for weight loss purposes. Evidence suggests that these strategies improve histological features of NAFLD and cardiometabolic risk factors [6,7,8].

Caloric restriction is the essential element of a nutrition intervention strategy for patients with NAFLD because it plays an important role in weight loss and improvement in liver steatosis and insulin sensitivity [9]. However, weight reduction and maintenance continue to be major challenges for many persons. It is possible that the modification of dietary components, such as the types and amounts of macronutrients consumed, with or without caloric restriction, may be a realistic and durable approach for patients with NAFLD [10]. The mechanism by which the consumption of specific nutrients influences this disease is not yet known. Previous studies reported varied proportions of macronutrients and dietary fiber being linked to insulin resistance and NAFLD development independent of total energy intake [11,12,13,14,15]. However, studies assessing dietary components in NAFLD patients compared with non-NAFLD patients or healthy controls showed discordant results [16,17]. This discrepancy may be related to the heterogeneity of liver disease and varying lifestyle behaviors.

To shed more light on this important evolving condition, the aim of this study was to prospectively examine for association between consumed nutrients and newly diagnosed NAFLD and insulin resistance in adults with different lifestyle behaviors. The results of this study will both improve our understanding of how NAFLD develops and facilitate the development of a more defined dietary strategy to treat and prevent NAFLD.

## 2. Materials and Methods

### 2.1. Participants

This cross-sectional study was conducted following the Declaration of Helsinki Ethical Principles, and the Institutional Review Board approved the study protocol. All participants were medical personnel recruited via flyers and posters that were distributed or hung at various locations around our medical center complex. Individuals who reported consuming alcohol less than 20 g/day in men and less than 10 g/day in women were invited to join this study from October 2016 to July 2017. All participants provided written informed consent to participate. Those matching one or more of the following criteria were excluded: being on a diet, performing moderate-to-vigorous exercise, being pregnant or lactating, having a self-reported chronic disease(s), and/or taking medications that induce weight loss or weight gain. Persons with previously diagnosed liver diseases, including fatty liver disease, chronic hepatitis B or C, autoimmune liver diseases, and hereditary liver diseases, were excluded.

### 2.2. Energy and Nutrient Intake Analysis

Participants were advised to maintain their normal eating habits during the food diary recording period. They took photos of their food intake over seven consecutive days using a smartphone camera held at a 45° angle and a one-arm distance from the food. Subjects captured photographic images of all food and beverages before and after consumption, and each meal was described/recorded in a paper-based food diary.

The study dietitian gathered information from a food diary and viewed the meal pictures with the highest clarity for the meal to estimate the type and the size of the food items using a photographic food atlas of food portion sizes [18]. For each meal consumed during each day of the seven-day assessment period, pictures recorded before consumption were analyzed for type and portion size of food items and, then after consumption, to evaluate the remaining amount of each food item. The amount of consumed food and drinks was calculated as the difference between the estimated portions served and the estimated leftovers. Dietary analysis was performed using INMUCAL-Nutrients software, version 3 (Institute of Nutrition, Mahidol University, Thailand), developed based on Thai food composition for energy, macronutrients, and selected micronutrients [19]. This program converts food intake into nutrients and calculates personal nutritional consumption each day [20,21,22,23]. The average daily dietary intake of all evaluated dietary components was calculated for each study participant.

### 2.3. Assessment of Hepatic Steatosis

Within a week after submitting their food diary, all participants underwent transient elastography with FibroScan^®^ 502 Touch (Echosens, Paris, France) using the M probe initially, and if the measurements were unreliable, with the XL probe to assess hepatic steatosis and fibrosis. All participants were fasted at least 3 h before the examination. The controlled attenuation parameter (CAP) was computed only when the liver stiffness measurement was valid, defined as an examination with 10 valid measurements with a success rate of at least 60% and an interquartile range/median ratio of 30% or lower. The median CAP and liver stiffness values are expressed as dB/m and kPa, respectively. The value of CAP at 288 dB/m or greater was used to establish the presence of hepatic steatosis, as defined by a magnetic resonance imaging proton density fat fraction ≥5% [24].

### 2.4. Clinical, Anthropometric, and Laboratory Evaluations

The socioeconomic status and lifestyle behavior of each participant was determined by age, sex, and healthcare profession. On the basis of healthcare profession, three groups were created: physician, nurse, and non-clinical staff. All participants underwent a complete clinical, anthropometric, and laboratory evaluation. Blood pressure was measured by an automatic sphygmomanometer with the subject seated after at least a 5 min rest. Anthropometry data, including weight, height, body mass index (BMI), and body circumference, were measured using standard procedures. Participants were categorized as normal (BMI 18.5–22.9 kg/m^2^), overweight (BMI 23–24.9 kg/m^2^), or obese (BMI ≥ 25 kg/m^2^) according to Asia-specific criteria [25]. Metabolic syndrome was diagnosed in individuals meeting three of the five following criteria: (1) hyperglycemia (fasting glucose ≥ 100 mg/dL) or previously diagnosed type 2 diabetes mellitus (fasting glucose ≥ 126 mg/dL or treatment with antidiabetic drugs); (2) hypertriglyceridemia (fasting triglycerides ≥ 150 mg/dL); (3) hypertension (systolic blood pressure ≥ 130 and/or diastolic blood pressure ≥ 85 mmHg); (4) low high-density lipoprotein cholesterol (HDL-C) (<40 mg/dL in men, <50 mg/dL in women); and, (5) central obesity (waist circumference ≥ 80 cm in women and ≥90 cm in men for Asians) [26].

Blood samples after an overnight fast were obtained for measuring plasma glucose, hemoglobin A1c (HbA1c), insulin, lipid profile (total cholesterol, low-density lipoprotein cholesterol (LDL-C), HDL-C, triglycerides), liver enzymes (aspartate aminotransferase (AST), alanine aminotransferase (ALT), alkaline phosphatase, gamma-glutamyl transferase (GGT), total bilirubin, and albumin), total 25-hydroxyvitamin D, platelet count, ferritin, high-sensitivity C-reactive protein (hs-CRP), and viral serology for hepatitis B and C infection. Biochemistry assays were performed using standardized methods. The homeostasis model for assessment of insulin resistance (HOMA-IR) method was used to evaluate fasting glucose and insulin levels [27]. HOMA-IR ≥2.5 was considered to indicate insulin resistance.

### 2.5. Statistical Analysis

Data were summarized by using descriptive statistics. Differences between groups were analyzed by the Student T-test or the Mann–Whitney U test for continuous variables and the χ^2^ test for categorical variables. Associations between the consumption of individual dietary items and the presence of NAFLD and insulin resistance were analyzed by logistic regression analysis. Individual components of dietary consumption were converted into categorical variables using their mean values among the entire cohort and recommended dietary components for a healthy adult (e.g., percentage of daily calories from carbohydrate, fat, and protein as well as grams of protein per kilogram of body weight). A dichotomization of dietary components from this step was used to analyze the univariate and multivariate logistic regression models, controlling for potential confounders, including age, sex, healthcare profession, and daily calorie intake. A *p*-value <0.05 was considered statistically significant in all analyses. Data were analyzed using SPSS Statistics version 18.0 (IBM Corporation, Armonk, NY, USA).

## 3. Results

### 3.1. Characteristics of the Study Population

After excluding subjects with a history of excessive alcohol use (*n* = 8), chronic hepatitis B virus infection (*n* = 1), and an incomplete food diary (*n* = 1), a total of 252 participants were included in our final analysis. The mean age of subjects was 37.6 ± 10 years, and their mean BMI was 23.8 ± 4.5 kg/m^2^. There was pronounced female predominance (81%), and seven (2.8%) subjects had type 2 diabetes. Among our cohort, 41 (16.3%) participants were newly diagnosed with NAFLD using a CAP of ≥288 dB/m. The mean liver stiffness measurement among subjects with NAFLD was 4.9 ± 0.9 kPa (range: 3.0–6.7). Vitamin D deficiency, defined as a serum total 25-hydroxyvitamin D less than 20 ng/dL, was detected in 135 (53.6%) members of the study cohort. Vitamin D deficiency was more prevalent in physician than in nurse and non-clinical staff (66.0% vs. 52.9% vs. 48.0%, respectively, *p* = 0.046).

Table 1 demonstrates the characteristics of the study population and a comparison between participants with and without NAFLD. Participants with NAFLD were more likely to be older, non-clinical staff and have a higher BMI, waist circumference, and waist-to-hip ratio. The proportion of persons with metabolic syndrome and its components was significantly higher in NAFLD subjects than in non-NAFLD subjects. In addition, participants with NAFLD had significantly higher levels of liver enzymes (AST, ALT, alkaline phosphatase, GGT), inflammatory biomarkers (ferritin, hs-CRP), glycemic markers (glucose, HbA1c, HOMA-IR), and lipoproteins (cholesterol, triglycerides, LDL-C), but lower HDL-C values than those without NAFLD. HOMA-IR ≥ 2.5 as an indicator of insulin resistance was more often observed in participants with NAFLD than in those without NAFLD (85.4% vs. 30.3%, *p* < 0.001).

### 3.2. Dietary Composition and NAFLD

The daily intake of macronutrients compared between those with and without NAFLD is shown in Table 2. Total energy intake and the proportion of carbohydrate, fat, and protein intake did not significantly differ between individuals with and without NAFLD. Daily intake of carbohydrate, fat, and protein components in participants with NAFLD expressed as both grams per day and percentage of total energy was comparable with those without NAFLD. Other dietary compositions, including saturated fat, cholesterol, different kinds of meat, starch, refined grain, refined sugar, vegetable, and fruit, were not significantly associated with NAFLD (Table 2).

**Figure 1:**
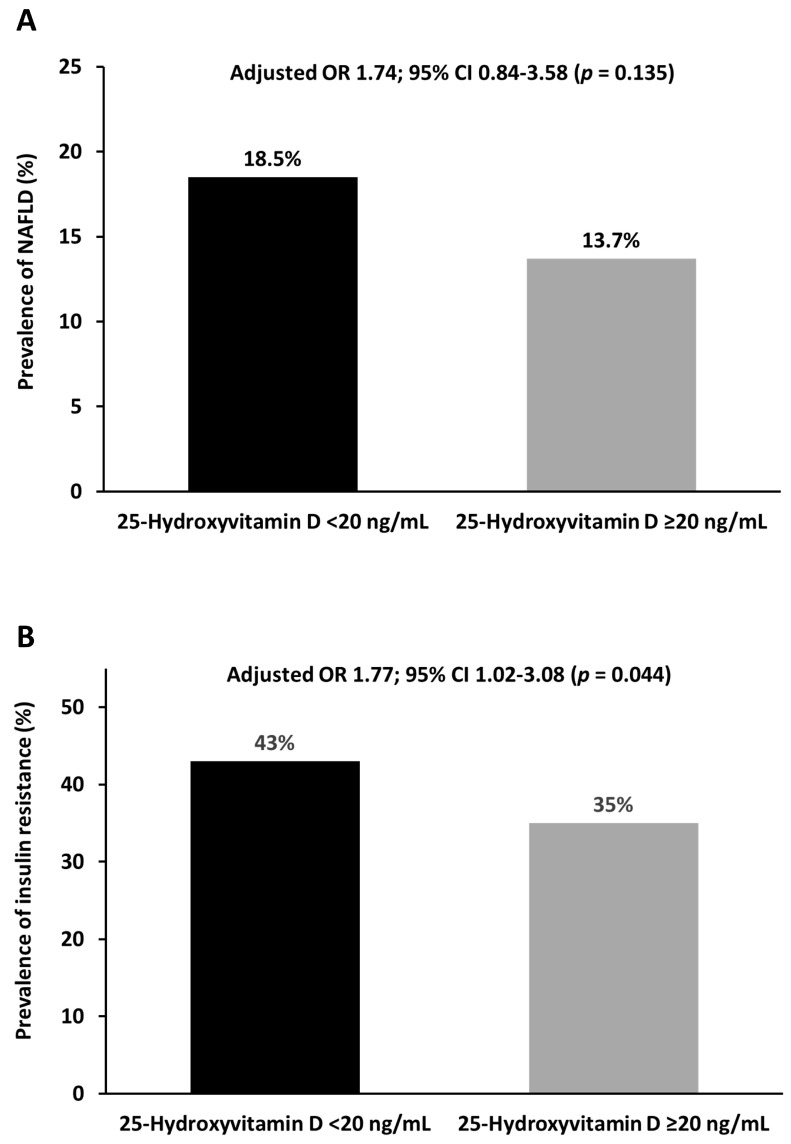
Adjusted odds ratio (OD) of vitamin D deficiency for (**A**) nonalcoholic fatty liver disease (NAFLD) and (**B**) insulin resistance after controlling for age, sex, healthcare profession, and daily calorie intake.

When expressed as grams of protein per kilogram of body weight, the amount of protein intake was significantly lower among participants with NAFLD than in those without NAFLD (0.84 ± 0.12 vs. 0.98 ± 0.36, *p =* 0.022). Since the dietary intake of 1.0 g of protein per kilogram of body weight per day is recommended for individuals with minimal physical activity [28], we used this recommended value of protein intake for the analysis. As shown in Table 3, subjects who consumed protein <1.0 g/kg daily (OR: 3.66, 95% CI: 1.41–9.52; *p* = 0.008) were more likely to have NAFLD than those consuming protein ≥1.0 g/kg daily even adjusting for age, sex, healthcare profession, and daily calorie intake. Interestingly, our analysis also revealed that participants with NAFLD consumed lesser full-fat dairy products than those without NAFLD (*p* = 0.013) (Table 2). When we treated full-fat dairy products as a categorical variable using a mean daily intake of 50 g in our total cohort, full-fat dairy product intake ≥50 g daily was inversely associated with the presence of NAFLD (OR: 0.42, 95% CI: 0.18–0.99; *p* = 0.047) when controlling for age, sex, healthcare profession, and daily calorie intake (Table 3). Individuals with NAFLD tended to consume lesser dietary fiber than those without NAFLD (7.40 ± 3.30 vs. 8.22 ± 3.83, *p* = 0.203) (Table 3). However, dietary fiber intake of at least 8 g daily was not associated with the presence of NAFLD (OR: 0.55, 95% CI: 0.25–1.20; *p* = 0.132) after adjusting for age, sex, healthcare profession, and daily calorie intake. Figure 1A shows that the prevalence of vitamin D deficiency was similar in participants with and without NAFLD (18.5% vs. 13.7%, *p* = 0.299). An adjusted model revealed no association between vitamin D deficiency and NAFLD (OR: 1.74, 95% CI: 0.84–3.58; *p* = 0.135).

### 3.3. Dietary Composition and Insulin Resistance

Given that insulin resistance is a hallmark and a causal factor of NAFLD, we assess whether commonly consumed nutrients correlate with the existence of insulin resistance, as shown in Table 2. Total energy intake, carbohydrate, fat, and protein expressed as both grams per day and percentage of total energy did not differ between participants with and without insulin resistance. Furthermore, there was no significant association between insulin resistance and other dietary components, such as starch, sugar, refined grain, vegetable, fruit, saturated fat, cholesterol, or different kind of meats. In contrast, the amount of protein intake per kilogram of body weight (*p* = 0.035), full-fat dairy product (*p* = 0.006), and dietary fiber intake (*p* = 0.020) were significantly associated with the presence of insulin resistance (Table 2).

When expressed as grams of protein per kilogram of body weight, the protein intake was significantly lower among participants with insulin resistance than in those without insulin resistance (Table 2). Individuals who consumed protein less than 1.0 g/kg daily (OR: 3.09, 95% CI: 1.59–6.05; *p =* 0.001) had a significantly higher probability of insulin resistance compared to those consuming protein ≥1.0 g/kg daily after adjusting for age, sex, healthcare profession, and daily calorie intake (Table 4). Furthermore, a full-fat dairy product consumption greater than 50 g daily was inversely associated with insulin resistance (OR 0.46, 95% CI: 0.25–0.82; *p* = 0.009) after adjusting for age, sex, healthcare profession, and daily calorie intake (Table 4). Though our population consumed an average amount of dietary fiber of only 8 g daily, consumption of dietary fiber of at least 8 g daily was associated with a protective role against insulin resistance (OR: 0.41, 95% CI: 0.22–0.74; *p* = 0.003) after adjusting for age, sex, healthcare profession, and daily calorie intake (Table 4). Interestingly, vitamin D deficiency was associated with increased odds for insulin resistance (OR: 1.77, 95% CI: 1.02–3.08; *p* = 0.044) after adjusting for age, sex, healthcare profession, and daily calorie intake (Figure 1B).

## 4. Discussion

Our results show that a low protein intake increases the odds for NAFLD and insulin resistance. In contrast, a high intake of full-fat dairy products and dietary fiber has been associated with a potential protective effect against NAFLD and insulin resistance. Notably, our subjects with NAFLD are characterized by a mild degree of hepatic inflammation with no/minimal liver fibrosis and insulin resistance-related comorbidities. These findings suggest that a diet deficient in protein, full-fat dairy products, and fiber may be involved in the early stage of NAFLD via the modulation of insulin sensitivity.

The role of diet in the pathophysiology of NAFLD is complex and extends beyond total energy intake. Although some investigators reported higher energy intake in patients with NAFLD than healthy controls [16], others found similar energy intake in both groups, with notable differences in dietary composition [17]. Several studies focused on detrimental associations between high animal protein intake and NAFLD [16,17,29,30]. To date, few studies have explored the association between insufficient protein intake and NAFLD risk. We identified significantly lower protein consumption in NAFLD participants than in non-NAFLD, independent of energy intake. Of note, protein intake was lower than the daily recommended level of at least 1.0 g/kg in 61.9% of our cohort, with a higher prevalence of 80.5% in subjects with NAFLD. Lipid accumulation in the liver following a low-protein diet may mediate via impaired peroxisomal, mitochondrial, and gut microbiota function [31]. Available evidence from animal models specific to the effects of protein malnutrition showed propionate and butyrate produced by gut bacteria to be associated with the risk of insulin resistance and NAFLD [31]. The data are consistent with our finding of a significant association between low protein intake and the chance of insulin resistance. To investigate causality, some researchers investigated the effects of supplementation with protein extract or amino acids on the low-protein-induced fatty liver disease. Specific amino acid supplementation can alleviate fatty liver disease in animal models fed with low-protein diets [32,33]. A high-protein diet reduced intrahepatic fat content without changing body weight in healthy humans compared to an isoenergetic high carbohydrate diet [34]. The existing data support that a low-protein diet may be responsible for lipid accumulation in the liver.

Emerging evidence in adults suggests that consuming full-fat dairy products is not related to the development of obesity or cardiovascular disease and might be beneficial [35,36,37]. Although full-fat dairy products contain more energy, many unique fatty acids in dairy fat, such as short-chain fatty acid (SCFA), conjugated linoleic acid, and palmitoleic acid, have hormone-like properties and show some potential health benefits [38]. The present study found a significant difference in full-fat dairy product intake between subjects with and without NAFLD, despite similar total and saturated fat consumption. Moreover, higher consumption of full-fat dairy products was inversely associated with insulin resistance, which also reduces NAFLD risk. This finding is aligned with a cross-sectional study by Kratz et al. that found that dairy fat improves glucose tolerance, possibly via a mechanism involving improved hepatic and systemic insulin sensitivity and decreased liver fat [39]. In our study, when analyzing full-fat dairy products based on the average intake among our entire study population, consuming ≥50 g/day of full-fat dairy products was associated with a lower chance of NAFLD even adjusting for age, sex, healthcare profession, and daily calorie intake. The observed associations suggest the potential benefits of full-fat dairy products on NAFLD.

The association between dietary fiber and NAFLD has not been extensively studied; however, much evidence has accumulated relative to the association between dietary fiber and metabolic dysfunction. In our study, consumption of dietary fiber was 2.4–17.9 g/day among subjects with NAFLD and 1.6–24.5 g/day in those without NAFLD. Furthermore, we found no significant difference in dietary fiber intake from any source (e.g., refined grain, fruit, or vegetable) between groups. Interestingly, despite almost all of our subjects consuming dietary fiber below the recommended level, we observed a significant association between fiber intake of at least 8 g per day and lower odds of insulin resistance after adjusting for age, sex, healthcare profession, and daily calorie intake. This finding is consistent with the results from prospective studies and clinical trials that showed high-fiber diets to be associated with improvement in insulin sensitivity and metabolic status [40,41,42,43]. Furthermore, the fermentation processes of dietary fiber in the colon that result in increased production of SCFA have been proposed as biological mechanisms involved in improved insulin sensitivity [44]. Hence, consuming foods naturally rich in fiber is particularly beneficial in those with NAFLD.

Macronutrient intake is not the only factor thought to play an essential role in developing NAFLD. Indeed, it has been indicated that aberrant serum levels of vitamins are common in individuals in the early stages of the disease. Low vitamin D status has become a common condition worldwide, and various studies have indicated its substantial associations with chronic diseases [45]. Sunlight exposure rather than nutrition has been reported as the primary source of vitamin D for the majority of the population [46]. However, lifestyle factors, such as extended working hours indoors and sun protection products have a negative impact on sufficient sun exposure. Therefore, a substantial proportion of the population, particularly those with a sedentary lifestyle, might rely on dietary vitamin D and body storage to maintain a healthy vitamin D status. Dietary sources of vitamin D comprise oil-rich fish, eggs, fortified foods (especially milk and dairy products), and small quantities of vitamin D in meat [47]. We found a high prevalence of vitamin D deficiency among our cohort, particularly physicians. Our multivariable analysis showed an independent association between vitamin D deficiency and insulin resistance but not with NAFLD. This finding is consistent with the results of several studies, indicating that vitamin D deficiency affects insulin secretion and may predispose to glucose intolerance [48]. Supplementations of vitamin D may alleviate insulin resistance. Further research is needed to assess the significant effects of vitamin D supplementation in insulin resistance.

The main strength of this study is that collecting dietary data over seven consecutive days makes our results representative of variable dietary habits under normal living conditions. Furthermore, the assessment of dietary intake using food photography and a food diary overcomes the inherent limitations of conventional approaches, such as self-administered questionnaires, which are susceptible to recall and reporting bias. Thus, our image-based dietary assessment should be a more accurate and reliable method for estimating energy and nutrient intake [49]. Some limitations of our study also need to be addressed. First, the cross-sectional design of this study does not allow causal inference. However, reverse causality is unlikely since all participants were unaware that they had NAFLD when collecting food data. Second, the sample size of this study restricts our ability to assess particular macronutrients that might be related to NAFLD and insulin resistance. Third, the nutritional data were collected from Asian adults comprising medical personnel with a preponderance of women, which may impact the generalizability across populations with differences in demographics, ethnicities, and lifestyle behaviors. Finally, we cannot exclude the possibility that undocumented/unreported dietary intake could confound associations between nutrients, NAFLD, and insulin resistance. Thus, we accounted for the socioeconomic status and lifestyle behavior (age, sex, and healthcare profession), as well as daily calorie intake in multiple regression analyses to compensate for the potential limitations.

## 5. Conclusions

The dietary behaviors of our subjects with NAFLD are characterized by a lower daily amount of protein consumption compared to those not found to have NAFLD despite the two groups having similar caloric intake and intake of other macronutrients. The observed protective effects of high intake of full-fat dairy products and dietary fiber on insulin resistance also support the relevance of these dietary components in the development of NAFLD. However, prospective longitudinal cohort studies with larger sample sizes are needed to confirm whether dietary intakes of certain food groups are associated explicitly with the risk of NAFLD and insulin resistance. Furthermore, before any definite dietary recommendations can be made for managing patients with NAFLD, intervention studies addressing the benefits of specific dietary modifications are necessary.

## Figures and Tables

**Table 1:** Demographic, clinical, and laboratory characteristics of the study population.

Characteristics	Total (*n* = 252)	NAFLD (*n* = 41)	No NAFLD (*n* = 211)	*p-*Value *
Age (years)	37.6 ± 10.0	40.9 ± 10.5	36.9 ± 9.8	0.019
Female sex, n (%)	204 (81.0%)	30 (73.2%)	174 (82.5%)	0.166
Healthcare profession				0.035
Physician	50 (19.8%)	5 (12.2%)	45 (21.3%)	
Nurse	104 (41.3%)	14 (34.1%)	90 (42.7%)	
Non-clinical staff	98 (38.9%)	22 (53.7%)	76 (36.0%)	
Body mass index (kg/m^2^)	23.8 ± 4.5	28.8 ± 4.5	22.8 ± 3.7	<0.001
Body mass index category				<0.001
Normal (18.5–22.9 kg/m^2^)	128 (50.8%)	1 (2.4%)	127 (60.2%)	
Overweight (23–24.9 kg/m^2^)	40 (15.9%)	7 (17.1%)	33 (15.6%)	
Obese (≥25 kg/m^2^)	84 (33.3%)	33 (80.5%)	51 (24.2%)	
Waist circumference (cm)	77.8 ± 10.8	88.3 ± 10.7	75.8 ± 9.6	<0.001
Waist/hip ratio	0.84 ± 0.07	0.87 ± 0.07	0.83 ± 0.07	<0.001
Systolic blood pressure (mmHg)	114 ± 14	124 ± 14	112 ± 13	<0.001
Diastolic blood pressure (mmHg)	74 ± 10	82 ± 10	73 ± 10	<0.001
Metabolic syndrome	20 (7.9%)	13 (31.7%)	7 (3.3%)	<0.001
Hypertension	11 (4.4%)	7 (17.1%)	4 (1.9%)	<0.001
Hypertriglyceridemia	30 (11.9%)	18 (43.9%)	12 (5.7%)	<0.001
Low HDL-C	39 (15.5%)	15 (36.6%)	24 (11.4%)	<0.001
Hyperglycemia/diabetes	46 (18.3%)	17 (41.5%)	29 (13.7%)	<0.001
Central obesity	81 (32.1%)	32 (78.0%)	49 (23.2%)	<0.001
AST (U/L)	20 ± 7	23 ± 12	18 ± 5	0.016
ALT (U/L)	19 ± 7	32 ± 26	16 ± 9	<0.001
Alkaline phosphatase (U/L)	58 ± 15	68 ± 18	56 ± 14	<0.001
GGT (U/L)	18 (13, 27)	34 (22, 54)	16 (12, 24)	<0.001
Total bilirubin (mg/dL)	0.55 ± 0.27	0.47 ± 0.20	0.56 ± 0.28	0.068
Albumin (g/dL)	4.57 ± 0.24	4.55 ± 0.28	4.57 ± 0.23	0.559
Platelet (× 10^9^)	281 ± 59	281 ± 57	281 ± 59	0.993
Glucose (mg/dL)	93 ± 18	107 ± 37	91 ± 10	0.006
Hemoglobin A1c, %	5.51 ± 0.67	6.04 ± 1.33	5.40 ± 0.35	0.004
HOMA-IR	2.09 (1.39, 3.14)	3.51 (2.74, 4.90)	1.87 (1.34, 2.76)	<0.001
Triglycerides (mg/dL)	77 (56, 109)	145 (109, 199)	71 (52, 97)	<0.001
Total cholesterol (mg/dL)	199 (171, 223)	207 (190, 244)	197 (167, 216)	0.003
HDL-C (mg/dL)	64 (52, 75)	49 (46, 56)	67 (55, 75)	<0.001
LDL-C (mg/dL)	112 (90, 135)	130 (108, 158)	111 (89, 133)	0.002
Total 25-hydroxyvitamin D (ng/mL)	19.6 ± 6.2	19.3 ± 5.4	19.7 ± 6.3	0.720
Ferritin (ng/mL)	82 (38, 146)	140 (54, 242)	77 (37, 127)	0.003
hs-CRP (mg/L)	0.97 (0.42, 2.28)	2.08 (1.24, 3.71)	0.76 (0.40, 2.16)	<0.001

Data presented as mean ± standard deviation, median (interquartile range), or number and percentage. * *p*-Value for comparison between patients with NAFLD and those without NAFLD. Abbreviations: ALT, alanine aminotransferase; AST, aspartate aminotransferase; GGT, gamma-glutamyl transferase; HDL-C, high-density lipoprotein cholesterol; HOMA-IR, homeostasis model assessment for insulin resistance; hs-CRP, high-sensitivity C-reactive protein; LDL-C, low-density lipoprotein cholesterol; NAFLD, nonalcoholic fatty liver disease.

**Table 2:** Composition of daily dietary consumption in relation to NAFLD and insulin resistance.

Dietary Composition	Total (*n* = 252)	NAFLD (*n* = 41)	No NAFLD (*n* = 211)	*p-*Value *	Insulin Resistance (*n* = 99)	No Insulin Resistance (*n* = 153)	*p-*Value **
Total energy intake (kcal)	1381 ± 328	1406 ± 381	1377 ± 317	0.601	1394 ± 367	1373 ± 300	0.632
Carbohydrate (g)	181.0 ± 46.7	181.9 ± 49.6	180.9 ± 46.3	0.896	181.5 ± 48.3	180.7 ± 45.9	0.905
Carbohydrate (% calorie)	52.6 ± 6.8	52.2 ± 6.5	52.6 ± 6.9	0.742	52.4 ± 6.1	52.7 ± 7.3	0.708
Fat (g)	47.7 ± 14.9	48.3 ± 17.5	47.5 ± 14.3	0.765	47.8 ± 15.8	47.6 ± 14.3	0.935
Fat (% calorie)	30.9 ± 5.4	30.5 ± 4.7	31.0 ± 5.6	0.598	30.6 ± 4.8	31.1 ± 5.8	0.499
Protein (g)	57.0 ± 20.0	60.9 ± 25.4	56.3 ± 18.7	0.263	59.4 ± 25.8	55.5 ± 15.0	0.175
Protein (% calorie)	16.5 ± 3.3	17.2 ± 3.8	16.3 ± 3.2	0.110	16.9 ± 3.9	16.2 ± 2.9	0.132
Protein (g per body weight kg)	0.96 ± 0.36	0.84 ± 0.12	0.98 ± 0.36	0.022	0.90 ± 0.43	1.00 ± 0.30	0.035
Saturated fat (g)	19.5 ± 31.0	22.8 ± 48.8	18.9 ± 26.4	0.622	20.3 ± 36.2	19.0 ± 27.3	0.774
Cholesterol (mg)	253.9 ± 123.7	267.0 ± 177.6	251.4 ± 110.7	0.592	256.4 ± 139.1	252.3 ± 113.1	0.806
Full-fat dairy (g)	49.7 ± 75.3	29.2 ± 49.4	53.1 ± 78.9	0.013	34.1 ± 59.7	59.0 ± 82.6	0.006
Red meat (g)	49.7 ± 36.6	60.3 ± 51.4	47.7 ± 32.7	0.135	53.9 ± 40.8	47.0 ± 33.5	0.163
White meat (g)	47.3 ± 34.9	51.0 ± 51.1	46.6 ± 31.0	0.593	49.6 ± 40.3	45.8 ± 31.0	0.422
Processed meat (g)	14.1 ± 18.6	14.3 ± 15.2	14.0 ± 19.2	0.914	13.7 ± 16.1	14.3 ± 20.1	0.788
Refined sugar (g)	50.2 ± 25.6	46.4 ± 23.8	52.1 ± 25.9	0.197	49.4 ± 25.3	52.3 ± 25.8	0.393
Starch (g)	294.0 ± 121.6	318.3 ± 144.0	289.3 ± 116.6	0.162	310.1 ± 129.3	283.6 ± 115.6	0.091
Fiber (g)	8.1 ± 3.8	7.4 ± 3.3	8.2 ± 3.8	0.203	7.4 ± 3.3	8.5 ± 4.0	0.020
Refined grain (g)	279.6 ± 124.4	302.1 ± 151.8	275.2 ± 118.3	0.289	294.0 ± 132.0	270.2 ± 118.7	0.138
Vegetable (g)	56.6 ± 43.4	56.2 ± 37.2	56.7 ± 44.6	0.950	55.7 ± 46.8	57.2 ± 41.2	0.778
Fruit (g)	85.3 ± 102.8	73.8 ± 101.0	87.6 ± 103.3	0.433	76.5 ± 98.3	91.0 ± 105.6	0.276

Note. Data presented as mean ± standard deviation. * *p*-Value for comparison between patients with NAFLD and those without NAFLD. ** *p*-Value for comparison between patients with insulin resistance and those without insulin resistance. Abbreviations: NAFLD, nonalcoholic fatty liver disease.

**Table 3:** Unadjusted and adjusted odds ratios of dietary composition associated with nonalcoholic fatty liver disease.

Dietary Composition	Univariate Analysis		Multivariate Analysis	
	Unadjusted Odds Ratio (95% CI)	*p-*Value	Adjusted Odds Ratio (95% CI) *	*p-*Value
Carbohydrate ≥ 60% of total energy	0.23 (0.03–1.73)	0.152	0.22 (0.03–1.74)	0.151
Fat ≥ 30% of total energy	0.66 (0.34–1.31)	0.234	0.69 (0.34–1.40)	0.302
Protein < 15% of total energy	0.66 (0.31–1.43)	0.296	0.59 (0.26–1.31)	0.194
Protein intake < 1.0 g/kg/day	2.95 (1.30–6.70)	0.010	3.66 (1.41–9.52)	0.008
Saturated fat ≥ 20 g/day	1.20 (0.49–2.94)	0.697	1.13 (0.42–3.05)	0.808
Cholesterol ≥ 254 mg/day	0.84 (0.42–1.68)	0.618	0.80 (0.36–1.80)	0.591
Full-fat dairy product ≥ 50 g/day	0.42 (0.19–0.96)	0.040	0.42 (0.18–0.99)	0.047
Red meat ≥ 50 g/day	1.01 (0.51–1.99)	0.985	1.09 (0.52–2.27)	0.829
White meat ≥ 47 g/day	0.94 (0.48–1.84)	0.851	0.91 (0.45–1.84)	0.793
Processed meat ≥ 14 g/day	1.05 (0.52–2.10)	0.899	1.16 (0.55–2.41)	0.699
Refined sugar ≥ 50 g/day	0.70 (0.35–1.38)	0.300	0.54 (0.25–1.19)	0.127
Starch ≥ 294 g/day	0.99 (0.51–1.95)	0.984	0.83 (0.38–1.79)	0.631
Dietary fiber intake ≥ 8 g/day	0.81 (0.41–1.61)	0.551	0.55 (0.25–1.20)	0.132
Refined grain ≥ 280 g/day	0.97 (0.50–1.91)	0.939	0.95 (0.44–2.03)	0.895
Vegetable (g) ≥ 57 g/day	0.91 (0.46–1.81)	0.793	0.89 (0.43–1.84)	0.758
Fruit ≥ 85 g/day	1.01 (0.50–2.01)	0.991	0.82 (0.38–1.75)	0.602

* Note: The multivariate model was adjusted for age, sex, healthcare profession, and daily calorie intake. Abbreviations: NAFLD, nonalcoholic fatty liver disease; CI, confidence interval.

**Table 4:** Unadjusted and adjusted odds ratio of dietary composition associated with insulin resistance.

Dietary Composition	Univariate Analysis		Multivariate Analysis	
	Unadjusted Odds Ratio (95% CI)	*p-*Value	Adjusted Odds Ratio (95% CI) *	*p-*Value
Carbohydrate ≥ 60% of total energy	0.43 (0.15–1.19)	0.104	0.41 (0.14–1.21)	0.106
Fat ≥ 30% of total energy	0.69 (0.42–1.15)	0.157	0.66 (0.38–1.13)	0.128
Protein < 15% of total energy	0.73 (0.42–1.27)	0.263	0.68 (0.38–1.23)	0.201
Protein intake < 1.0 g/kg/day	2.19 (1.27–3.78)	0.005	3.09 (1.59–6.05)	0.001
Saturated fat ≥ 20 g/day	0.89 (0.43–1.81)	0.738	0.88 (0.40–1.94)	0.750
Cholesterol ≥ 254 mg/day	0.83 (0.49–1.39)	0.481	0.75 (0.41–1.37)	0.346
Full-fat dairy product ≥ 50 g/day	0.48 (0.28–0.85)	0.011	0.46 (0.25–0.82)	0.009
Red meat ≥ 50 g/day	1.19 (0.71–2.00)	0.509	1.40 (0.80–2.47)	0.239
White meat ≥ 47 g/day	1.24 (0.75–2.06)	0.405	1.22 (0.72–2.09)	0.459
Processed meat ≥ 14 g/day	1.13 (0.67–1.91)	0.658	1.13 (0.65–1.98)	0.656
Refined sugar ≥ 50 g/day	0.72 (0.43–1.19)	0.200	0.58 (0.32–1.05)	0.073
Starch ≥ 294 g/day	1.34 (0.81–2.24)	0.254	1.41 (0.78–2.55)	0.254
Dietary fiber intake ≥ 8 g/day	0.54 (0.32–0.92)	0.022	0.41 (0.22–0.74)	0.003
Refined grain ≥ 280 g/day	1.31 (0.79–2.18)	0300	1.45 (0.81–2.59)	0.207
Vegetable (g) ≥ 57 g/day	0.90 (0.54–1.51)	0.701	1.01 (0.58–1.75)	0.985
Fruit ≥ 85 g/day	0.69 (0.40–1.17)	0.169	0.60 (0.33–1.09)	0.094

* Note: The multivariate model was adjusted for age, sex, healthcare profession, and daily calorie intake. Abbreviations: NAFLD, nonalcoholic fatty liver disease; CI, confidence interval.

## Data Availability

The datasets used in this study are available from the corresponding author.
